# Streamlined Full-Length Total RNA Sequencing of Paraformaldehyde-Fixed Brain Tissues

**DOI:** 10.3390/ijms25126504

**Published:** 2024-06-13

**Authors:** Bingqing Ji, Jiale Chen, Hui Gong, Xiangning Li

**Affiliations:** 1 Britton Chance Center for Biomedical Photonics, Wuhan National Laboratory for Optoelectronics, Huazhong University of Science and Technology, Wuhan 430074, China; jibingqing@hust.edu.cn (B.J.); chenjiale96@hust.edu.cn (J.C.); huigong@mail.hust.edu.cn (H.G.); 2 MoE Key Laboratory for Biomedical Photonics, Department of Biomedical Engineering, Huazhong University of Science and Technology, Wuhan 430074, China; 3 Research Unit of Multimodal Cross Scale Neural Signal Detection and Imaging, HUST-Suzhou Institute for Brainsmatics, JITRI, Chinese Academy of Medical Sciences, Suzhou 215125, China; 4 Key Laboratory of Biomedical Engineering of Hainan Province, School of Biomedical Engineering, Hainan University, Haikou 570228, China

**Keywords:** paraformaldehyde fixation, cross-link reversal, RNA sequencing, full-length transcripts, protein-coding genes, non-coding RNAs

## Abstract

Paraformaldehyde (PFA) fixation is the preferred method for preserving tissue architecture for anatomical and pathological observations. Meanwhile, PFA reacts with the amine groups of biomolecules to form chemical cross-linking, which preserves RNA within the tissue. This has great prospects for RNA sequencing to characterize the molecular underpinnings after anatomical and pathological observations. However, RNA is inaccessible due to cross-linked adducts forming between RNA and other biomolecules in prolonged PFA-fixed tissue. It is also difficult to perform reverse transcription and PCR, resulting in low sequencing sensitivity and reduced reproducibility. Here, we developed a method to perform RNA sequencing in PFA-fixed tissue, which is easy to use, cost-effective, and allows efficient sample multiplexing. We employ cross-link reversal to recover RNA and library construction using random primers without artificial fragmentation. The yield and quality of recovered RNA significantly increased through our method, and sequencing quality metrics and detected genes did not show any major differences compared with matched fresh samples. Moreover, we applied our method for gene expression analysis in different regions of the mouse brain and identified unique gene expression profiles with varied functional implications. We also find significant dysregulation of genes involved in Alzheimer’s disease (AD) pathogenesis within the medial septum (MS)/vertical diagonal band of Broca (VDB) of the 5×FAD mouse brain. Our method can thus increase the performance of high-throughput RNA sequencing with PFA-fixed samples and allows longitudinal studies of small tissue regions isolated by their in situ context.

## 1. Introduction

PFA is one of the most widely used tissue fixative reagents in academic and medical settings. The monomeric PFA reacts with the amine groups of proteins and nucleic acids to form chemical bonds within the tissue, thereby stabilizing the tissue architecture and protecting biomolecules from degradation [1,2,3]. As a consequence, PFA fixation offers a high degree of preservation of morphological detail while preserving biomolecules in situ. In addition, because the monomeric PFA is a tiny molecule, PFA is highly permeable in tissues [4] and is slow to react with biomolecules [5], rendering it uniquely suited for uniform fixation of large-scale tissues [6], such as the whole mouse brain tissue. Thus, a vast number of PFA-fixed mouse brain biospecimens are used for the study of anatomical [7] and pathological features [8], offering great prospects for gene expression research to further characterize the molecular underpinnings. However, PFA fixation negatively affects RNA accessibility due to RNA being cross-linked to adjacent biomolecules by the addition of methylol groups and methylene bridge formation [9,10,11]. These chemical modifications on the RNA molecules inhibit primer annealing and elongation during reverse transcription and PCR amplification, resulting in low RNA sequencing sensitivity [12].

Traditionally, gene expression analysis of PFA-fixed biospecimens has involved using short nucleic acid probes to hybridize with target RNAs [13]. These methods depend on the availability of previously known molecular markers [14] and thus were of low throughput until the recent development of methods for high-throughput ISH analysis [15,16]. Lately, several high-throughput sequencing-based approaches have been developed for accurate gene expression analysis of PFA-fixed biospecimens. One of the methods for sequencing-based analysis is DBiT-seq [17,18], which has demonstrated its value when applied to thin tissue sections that were 4% PFA-fixed for 20 min in mouse embryos. Other methods called FD-seq [19] and FRISCR [20] were shown to be compatible with 4% PFA-fixed cells, but their fixed time is limited to 15 min. The RNA obtained by these methods showed excellent quality and quantity, and the detected genes and gene expression levels were close to the unfixed samples. Because the cross-linking reaction of PFA is relatively slow, it presumably does not complete during a short fixation period. A high-throughput sequencing method [21] that combines cross-link reversal and mRNA capture by oligo(dT) has been shown to work with PFA fixation for 24 h in human lung and kidney organoids. PFA fixation was considered to be completely finished within 24 h [22]. However, it is not sensitive to transcript length, which is a confounder for whole-transcript RNA sequencing, and it cannot detect non-polyadenylated transcripts. Furthermore, RNA sequencing strategies have been developed for application on formalin-fixed paraffin-embedded (FFPE) samples [23,24], which typically involve prolonged fixation. Paraffin embedding has a great adverse impact on RNA [25,26]. Consequently, gene expression data from FFPE samples are significantly different from those of unfixed samples and do not support gene expression profiling for the entire transcriptome. In summary, despite many technical advances, reliable sequencing and accurate gene expression quantitation in prolonged PFA-fixed tissue continue to be challenging.

Here, we present a method of gene expression analysis in PFA-fixed samples, including the precise isolation of specific regions in the PFA-fixed brain tissue, RNA recovery, library construction, and RNA sequencing. We compared the yield and quality of recovered RNA, sequencing quality metrics, and detected genes between PFA-fixed and matched fresh samples. These analyses indicate that our proposed method introduces little bias and yields gene expression data similar to that from fresh tissue. By applying our method to perform gene expression profiling in MS/VDB, the hypoglossal nucleus (XII), and the medial prefrontal cortex (mPFC) regions isolated from the PFA-fixed mouse brain, we have found significantly diverse gene expression patterns with varied functional implications. We then utilized our method to study the MS/VDB region with AD-related pathological features in the PFA-fixed 5×FAD mouse brain. We discovered that molecular signatures change associated with pathological features compared with the control mouse. We anticipate that our method will be a valuable tool to understand biological systems at multiple levels.

## 2. Results

### 2.1. Development of an RNA Sequencing Method in PFA-Fixed Samples

Recovery of PFA-fixed RNA for high-throughput sequencing is achieved by removing cross-links as well as sequencing library construction using random primers. The protocol used for PFA-fixed samples differs from the established fresh tissue workflow in several aspects, allowing for cross-link reversal and eliminating the need for a fragmentation process.

**Figure 1:**
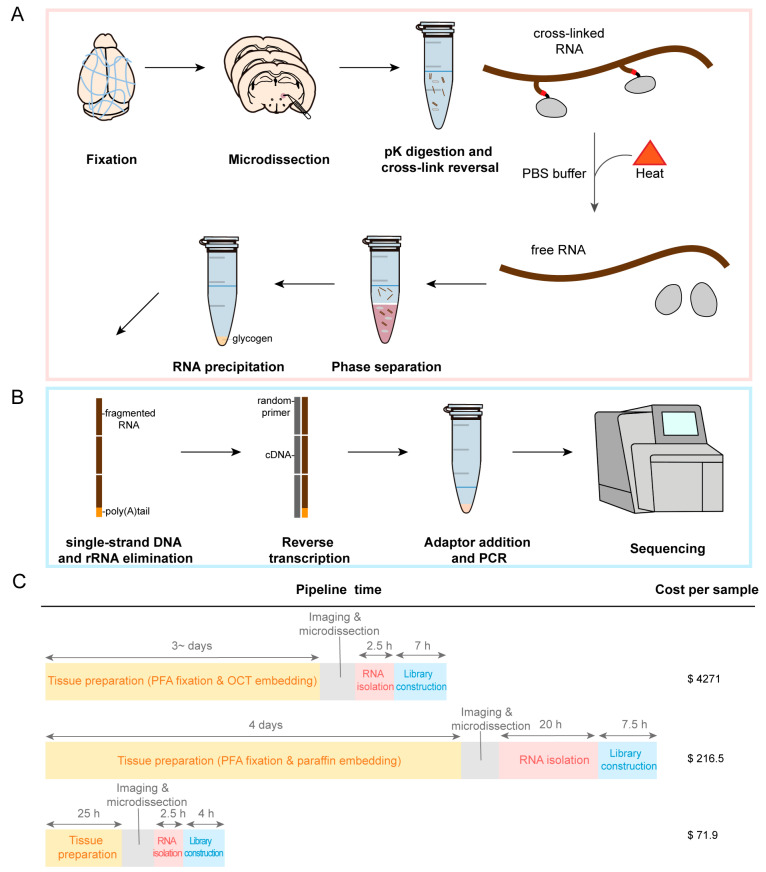
Schematic flow of RNA sequencing in PFA-fixed brain tissue. (**A**) Sectioning and microdissection of regions of interest from PFA-fixed brain tissue; RNA is recovered using heat cross-link reversal with proteinase K in PBS buffer, followed by phenol–chloroform extraction and precipitation of total RNA with ethanol in the presence of sodium acetate and glycogen. (**B**) RNA from PFA-fixed brain tissue is enriched by depleting rRNA from total RNA. The fragmented RNA from the PFA-fixed sample is converted directly to cDNA without fragmentation. The 1st strand of cDNA is synthesized with random RT primers. The single-stranded cDNA is then converted to double-stranded cDNA, adaptors are added, and a PCR reaction is performed. Finished libraries are sequenced by a synthesis procedure using an Illumina Genome Analyzer. (**C**) Comparison of our method with selected RNA-seq used in different tissue preparation methods [21,23]. The time required for tissue preparation, RNA isolation, and library construction is indicated by the double arrow lines at the top. Cost per sample includes all reagents but not consumables (tubes, pipet tips).

**Figure 2:**
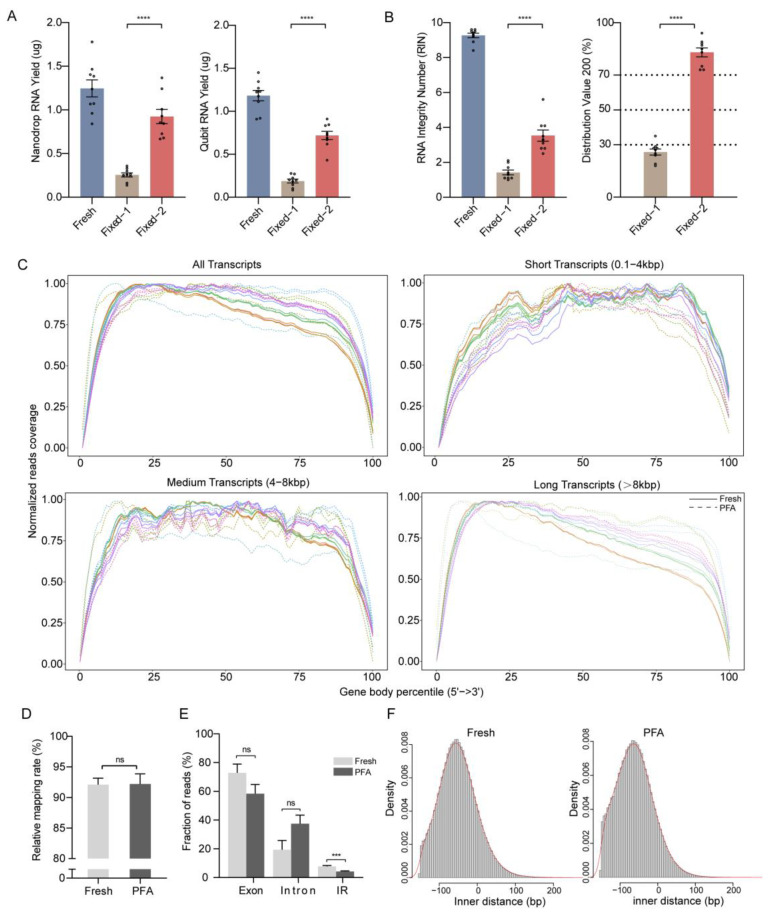
Benchmarking and validation of our method in RNA and sequencing library quality metrics. (**A**) Total RNA yield as assessed by Nanodrop spectrophotometer and Qubit fluorometer. (**B**) RIN and DV200 as measured by Bioanalyzer of RNA isolated from fresh brain tissue, of RNA isolated from PFA-fixed brain tissue without heat cross-link reversal using proteinase K in PBS buffer (labeled “Fixed−1”), and of RNA isolated from PFA-fixed brain tissue after heat cross-link reversal using proteinase K in PBS buffer (labeled “Fixed−2”). (**C**) Reads coverage along the normalized transcript length. Coverage plots along the normalized transcript length for all transcripts and for three different transcript length categories (0.1−4 kbp, 4−8 kbp, >8 kbp) are shown to determine the effect of transcript length on the coverage along the entire transcript. One colorful line represents one sample. The tissue preparation types are shown with different line styles (Fresh: solid; PFA: dashed). (**D**) The relative amount of mapped vs. unmapped reads. (**E**) The fraction of reads mapped to exon, intron, or intergenic region. (**F**) Paired-end inner distance distributions. Negative values correspond to overlapping paired-end reads. Data are presented as mean ± SEM. *n* = 9 technical replicates for the PFA and Fresh groups. *** *p* < 0.001, **** *p* < 0.0001; n.s., not significant. Two-sided *t*-tests.

First, the tissue was enzymatically permeabilized with protease K, followed by heat cross-link reversal and RNA purification (Figure 1A). Protease K was included in our protocols to boost the solubilization of the fixed tissue. Cross-link reversal was performed by heat-induced retrieval at 70 °C with PBS buffer. After cross-link reversal, the RNA was isolated from other compounds by phenol–chloroform extraction, and it was precipitated using ethanol in the presence of sodium acetate and glycogen. Next, sequencing libraries were prepared by single-strand DNA and rRNA depletion for transcriptome enrichment and reverse transcription with random primers (Figure 1B). rRNA is considered transcriptome noise, which makes up 80% of total RNA and is the predominant type of total RNA. rRNA removal allows for more efficient access to biological information. The RNA integrity number (RIN) of the fixed tissues was much lower than that of the total RNA extracted from the fresh brain tissues (RIN was 9.3 ± 0.1, Figure 2B), demonstrating that removing cross-links invariably resulted in total RNA fragmentation. Therefore, all RNA fragments except rRNA were reverse-transcribed using random hexamers rather than oligo-dT.

Our workflow was compared step by step with similar protocols (Table S1). The short number of steps allows a batch of samples to be prepared in ~32 h from tissue preparation to final library acquisition. We profiled certain regions of the mouse brain using only basic laboratory equipment with a total cost of about USD 72 per sample (Figure 1C). PFA-fixed agarose-embedded tissue sections are easier to obtain than optimal cutting temperature compound (OCT) and paraffin-embedded tissue sections, while identification of small regions by imaging in PFA-fixed tissue sections is more precise and reliable than in fresh tissue sections. Because of the efficiency of cross-link reversal with PBS buffer, it is sensitive to very small amounts of starting tissue. The experimental requirements of phenol–chloroform extraction combined with ethanol precipitation are low compared with RNA purification methods based on silica adsorption and solid phase reverse immobilization. The total RNA is fragmented before library construction, omitting the standard step of fragmentation. Furthermore, because our method reads all fragments of each transcript except rRNA, it can detect the full length of transcripts with random primers and report information on non-polyadenylated and non-coding transcripts. Thus, our method allows simple and efficient quantification of the abundance of various genes.

### 2.2. Validation of Method Compared to Reference Fresh Samples

We validated the accuracy and sensitivity of our method by comparing it side-by-side with intact RNA and sequencing data from matched fresh samples. In order to assess the yield of isolated RNA after cross-link removal, we used absorbance and dye-based quantitation. Comparatively to fixed tissue of the same size without removing cross-links, RNA yield, as assessed by a Nanodrop spectrophotometer, increased from 0.26 ± 0.02 µg to 0.92 ± 0.08 µg, and the RNA recovery rate increased from 22 ± 2.8% to 76 ± 5.4%. Similarly, RNA yield, as measured by a Qubit fluorometer, went from 0.19 ± 0.02 µg to 0.72 ± 0.05 µg, and the RNA recovery rate rose from 16 ± 2.4% to 62 ± 5.3% (Figure 2A). To assess the quality of RNA, RIN and distribution value 200 (DV200) were utilized. RIN increased from 1.4 ± 0.1 to 3.5 ± 0.3 and DV200 from 26 ± 2% to 83 ± 3% (Figure 2B). This suggests that additional steps of cross-link removal during RNA isolation can improve the RNA yield and quality of PFA-fixed tissue. Agarose does not penetrate into tissues, immersion and solidification time is short, and our results confirm that agarose embedding does not reduce the quantity and quality of RNA (Figure S1).

RNA performance in enzymatic assays of library construction was improved after cross-link removal. The coverage plots over normalized transcript length for all PFA and fresh samples are used to identify problems that may have occurred during the library construction. With increasing transcript length, we observe a more pronounced 5′ bias with long transcripts (>8 kbp) in both PFA and fresh samples; however, all samples have uniform coverage distributions, independent of the transcript length for short and medium transcripts. No significant 3’ bias in the coverage of all transcripts was found (Figure 2C). The percentage of alignable reads was 92.1 ± 1.04% in the Fresh libraries. Of reads from the PFA libraries, 92.2 ± 1.67% were alignable, which showed that the percentage of reads mapping was similar to that of the Fresh libraries (Figure 2D). These results collectively suggest that our library construction method for PFA samples effectively eliminates the 3′ bias expected from the heavily fragmented RNA that utilizes oligo-dT capture and amplification. Among them, the ratio of reads mapped to exon and intron regions in the PFA group was 58.4 ± 6.39% and 37.5 ± 5.97%, respectively. In the Fresh group, the ratio of reads mapped to the exon and intron regions was 72.9 ± 6.07% and 19.3 ± 6.45%, respectively. There was a small but significant decrease in reads mapping to intergenic regions in the PFA group (4.2 ± 0.43%) relative to the Fresh group (7.8 ± 0.58%) (Figure 2E). The PFA and Fresh groups showed a higher percentage of reads mapped to exons. Visualization of single-gene reads from the PFA and Fresh groups also indicates that a large fraction of reads mapped to the exon regions (Figure S2). A good proxy to evaluate the RNA molecule length is to calculate the paired-end inner distances for each RNA sequencing experiment. The inner distance of the fragmented RNA in the PFA group was discovered to be −60 ± 49 bp, and the artificial fragmentation of the intact RNA in the Fresh group was found to be −50 ± 50 bp. The distribution of inner distances was comparable. In accordance with this, the peak lengths of the insert sizes were 240 and 250 bp, respectively (Figure 2F). The fragmented RNA from the PFA group and the artificially fragmented RNA from the Fresh group exhibited very similarly. This indicates that the sequencing quality metrics of the PFA group did not show any major differences compared with the Fresh group.

**Figure 3:**
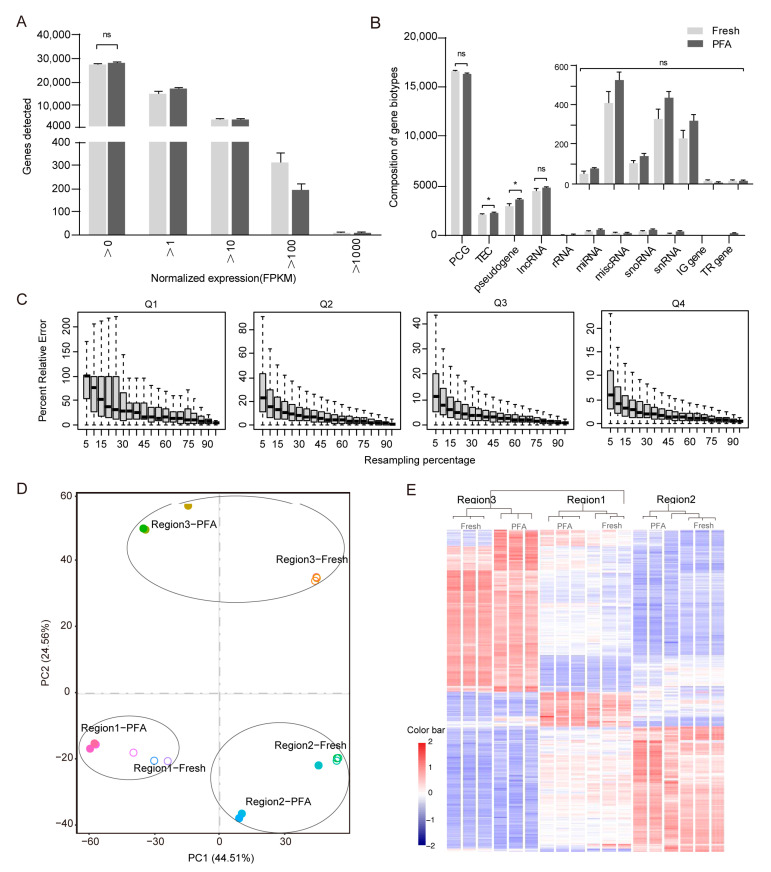
Comparison between PFA fixation and fresh sample shows similar gene recovery with our method. (**A**) PFA fixation and matched fresh samples show the number of unique genes detected at each FPKM threshold. (**B**) Composition of detected gene biotypes in the two types of samples. Data in a and b are shown as mean ± SEM. *n* = 9 technical replicates for the PFA and Fresh groups. * *p* < 0.05; n.s., not significant. Two-sided *t*-tests. (**C**) The effects of sequencing depth on the number of detected genes by resampling a series of subsets from total reads and checking if the FPKM values were stable or not in terms of gene expression estimation. A total of 20 FPKM values were calculated using 5%, 10%, …, 95%, and 100% of total reads; real FPKM value was estimated from total reads. (**D**) Principal component analysis of all genes across the PFA and Fresh groups from three regions. The solid circle represents the PFA group, and the hollow circle represents the Fresh group. (**E**) Hierarchical clustering based on the intersection of DEGs for all 18 samples. For data in D and E, *n* = 3 technical replicates for all samples.

To determine whether our method can generate enough information, we selected RNA sequencing data from multiple regions of PFA-fixed brain tissue and compared them to those of the same regions of matched fresh brain tissue. The number of genes detected in the PFA group was comparable to that of the Fresh group across all samples (28,535 ± 492 genes in the PFA group compared to 27,098 ± 721 genes in the Fresh group). The distribution of normalized expression levels in the PFA group was also similar to that in the Fresh group. The number of genes in the PFA group and Fresh group accounted for 84.5 ± 0.43% and 82.9 ± 0.71% of the total number of genes in the fragments per kilobase per million mapped reads (FPKM) value range of 0~10, and there was no statistically significant difference between them (Figure 3A). In the PFA group, there were 16,432 ± 81 PCGs (protein-coding genes), 2253 ± 55 TEC (to be experimentally confirmed), and 3554 ± 126 pseudogenes. The amount of lncRNA (long non-coding RNA) was 4711 ± 134; 79 ± 8 for rRNA, 526 ± 42 for miRNA, 146 ± 10 for miscRNA, 442 ± 35 for snoRNA, and 324 ± 28 for snRNA, respectively. In addition, there were 17 ± 2 IG genes (immunoglobulin genes that undergo somatic recombination) and 12 ± 1 TR genes (T cell receptor genes that undergo somatic recombination), separately. Most of the detected genes were annotated as protein-coding in both groups. Except for the fact that the PFA group included a greater percentage of TEC and pseudogenes, there was no discernible difference in the number of genes between various biotypes compared with the Fresh group (Figure 3B). To determine the effect of sequencing depth on the number of detected genes, we randomly resampled the sequencing reads of the PFA group and found that the relative error rate is nearly zero with little variation starting from the random resampling of 50% of the total reads (Figure 3C). This demonstrated that the sequencing depth was saturated, and the number of genes detected was real and reliable.

We further compared our expression profiles with fresh samples. Principal component analysis of all genes from three regions of the PFA/fresh pairs revealed that Region1 groups and Region2 groups were always segregated in two different areas of the 2D plots in the first dimension (PC1), while Region3 groups were segregated in a different area in the second dimension (PC2). However, it is important to note that there is a minor separation between different tissue preservation techniques in each group of brain regions (Figure 3D). A similar pattern of clustering was observed in the hierarchical cluster analysis performed using the intersection of DEGs of all comparison groups. The expression profiles clustered preferentially based on region rather than the method used to preserve the brain tissue, i.e., Fresh vs. PFA (Figure 3E). The relative expression levels of the detected genes were well correlated (Pearson’s correlation coefficient was ~0.9168 except in one instance where it was 0.6761) between the Fresh and PFA groups after normalization by FPKM and transformation to log10 count (Figure S3). We also assessed the technical repeatability by analyzing three technical replicates of the PFA-fixed sample with our method. We found that the three replicates showed strong agreement in terms of the relative gene expression level (Figure S4).

Taken together, these results demonstrate that our method is a reliable method for the whole-transcriptome analysis of PFA-fixed tissue. The performance of RNA sequencing is maintained with fixed tissue when our method is utilized, and our method shows comparable performance to the standard RNA sequencing protocol used for fresh, unfixed tissue.

### 2.3. The Diversity of Gene Expression Profiling in Different Regions of the Mouse Brain

**Figure 4:**
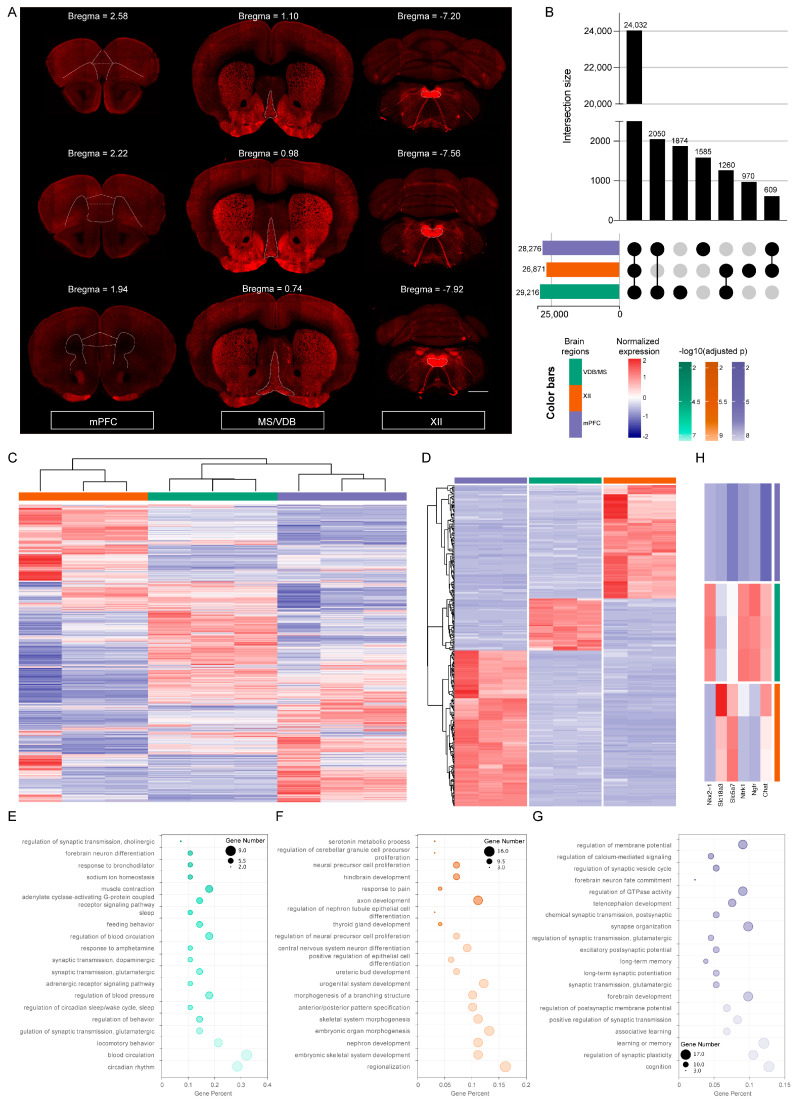
Identification of gene expression differences in MS/VDB, XII, and mPFC with our method. (**A**) Representative coronal sections showing the distribution of cholinergic neurons in MS/VDB, XII, and mPFC regions of ChAT-ires-Cre:Ai14 mouse brain. The closed dotted lines represent the outlines of the three brain regions. Scale bars, 1000 µm. (**B**) The number of shared and unique sets of expressed (FPKM ≥ 0.1) genes in three brain regions. (**C**) Hierarchical clustering analysis of gene expression profiles of all 9 samples with 18,552 genes. (**D**) Expression profiles of 315 brain-region-specific genes across all samples. (**E**) Top 20 GO enrichment terms (adjusted *p* < 0.05) of the brain-region-specific expressed genes in MS/VDB. (**F**) Top 20 GO enrichment terms (adjusted *p* < 0.05) of the brain-region-specific expressed genes in XII. (**G**) Top 20 GO enrichment terms (adjusted *p* < 0.05) of the brain-region-specific expressed genes in mPFC. (**H**) Markers for cholinergic neurons differentially expressed between MS/VDB, XII, and mPFC. Color bars for panels **B** to **H** are located at the bottom of panel **B**. In panels **C** and **D**, each column represents a sample, whereas each row represents a gene. In panel **H**, each row represents a sample, while each column represents a marker for cholinergic neurons. The color bar in each cluster symbolizes the brain region: green for MS/VDB, orange for XII, and purple for mPFC. In panels **E** to **G**, each panel corresponds to a brain region with the dots colored according to the adjusted *p*-values.

According to the distribution characteristics of specially labeled neurons in specific brain regions of transgenic mice, we performed a microdissection of fixed ChAT-ires-Cre:Ai14 mouse brain sections under the confocal microscope (Figure 4A). Gene expression profiles of the MS/VDB, XII, and mPFC were obtained by our method. The cortical regions, subcortical regions, and brainstem motor nuclei are all involved. On average, 26,552 (70.8%) of the 37,514 genes were defined as expressed (FPKM ≥ 0.1). Differences among brain regions in the number of genes expressed were observed. The MS/VDB expressed the most genes (29,216), followed by mPFC (28,276), whereas the XII expressed the least number of genes (26,871). Comparing the above regions of interest (ROIs) among themselves, we found a shared signature comprising 24,032 genes, with each ROI having unique sets of genes (Figure 4B). We also identified 18,552 genes ubiquitously expressed (FPKM ≥ 0.1 across all samples), which should play a vital role in the basic biological functions of cells, 13,561 and 2376 of which belong to PCGs and ncRNA, respectively. Hierarchical cluster analysis (Figure 4C) of all samples demonstrated that different brain regions exhibited unique gene expression profiles. Additionally, we carried out distinct pairwise comparison studies for PCGs or ncRNA alone (Figure S5). The results showed that the expression pattern of PCGs was very similar to that of the whole set of genes, whereas the ncRNA genes displayed less divergence among brain regions. MS/VDB and mPFC had a relatively high number of over-expressed ncRNA genes compared with XII.

We also identified brain-region-specific genes, which we characterize as having an expression level that is more than eight-fold higher in a given brain region than in any other region (Table S2). Totally, we identified 315 brain-region-specific genes, and the number of brain-region-specific genes varied from region to region. mPFC expressed the most brain-region-specific genes (153), closely followed by XII (111) and MS/VDB (51) (Figure 4D). Functional enrichment analysis was used to reveal the biological meaning behind these brain-region-specific genes. Generally, we observed that these genes were enriched in different GO terms and were highly correlated with particular biological processes or the development of a specific brain region (Table S3). Among them, the MS/VDB-specific genes were mainly associated with circadian rhythm, sleep, feeding behavior, glutamatergic synaptic transmission, positive regulation of cholinergic synaptic transmission, and forebrain neuron differentiation (Figure 4E). The XII-specific genes were involved in the biological processes of skeletal system morphogenesis and morphogenesis of a branching structure (Figure 4F). This might be related to the fact that the XII innervates the tongue’s intrinsic and extrinsic muscles, as well as the musculus hyoideus [27,28]. Furthermore, processes involved in central nervous system neuron differentiation, axon development, neural precursor cell proliferation, and hindbrain development were identified in XII-specific genes (Figure 4F). Genes specifically expressed in mPFC were mainly enriched in associative learning, long-term memory, cognition, and forebrain and telencephalon development. GO terms associated with chemical synaptic transmission biological processes, such as regulation of synaptic plasticity, long-term synaptic potentiation, and excitatory postsynaptic potential, were also identified (Figure 4G). Collectively, these findings showed that three brain regions have significantly different gene expression patterns with distinct functional implications.

Finally, we investigated *Chat* and other cholinergic neuron markers. *Ngfr* [29] encodes the nerve growth factor (NGF) receptor, and *Ntrk1* [30] encodes TrkA (tropomyosin receptor kinase A, a receptor for NGF), which is essential for the development of cholinergic neurons. *Slc5a7* [31] encodes the presynaptic choline transporter (CHT), which is a key determinant of synapse acetylcholine synthesis and release; *Slc18a3* [32] encodes vesicular acetylcholine transporter (VAChT), and *Nkx2-1* [33,34] encodes transcription factors necessary for the development of cholinergic neurons. It was found that the expression abundance and the number of cholinergic-neuron-related markers were higher in MS/VDB and XII compared to mPFC (Figure 4H), which was consistent with the actual distribution of cholinergic neurons in these three regions (Figure 4A). Such molecular studies after anatomical observation may accelerate our understanding of biological systems at multiple levels.

### 2.4. The Molecular Signatures Associated with Pathological Features in the AD Mouse Model

**Figure 5:**
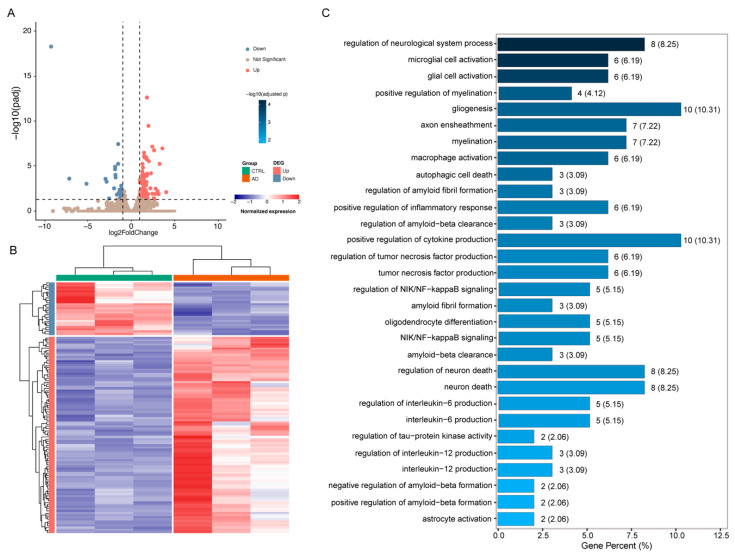
DEGs and their associated biological processes in 5 xFAD mouse model. (**A**) DEGs between AD and CTRL. The dashed line indicates the threshold of the padj and FoldChange.The DEGs with |log_2_(FoldChange)| > 1 and padj < 0.05 are colored in red (up-expressed DEGs in AD mouse) and blue (down-expressed DEGs in AD mouse), respectively. (**B**) The DEG composition between AD (orange) and CTRL group (green). (**C**) Top 30 GO enrichment biological processes (adjusted *p* < 0.05) of the DEGs between AD and CTRL. Each bar in panel (**C**) represents the number (percent) of genes in the biological process, while the shades of blue represent the adjusted *p*-values.

We further applied this method to generate gene expression data in the MS/VDB region of mouse brains with pathological characteristics of AD to assess transcriptome differences from control mice. By comparing their gene expression profiles, we identified 118 differentially expressed genes (DEGs) (|log_2_(FoldChange)| > 1 and adjusted *p* < 0.05) between the AD and CTRL groups (Figure 5A). Among them, 93 and 25 genes were up- and down-expressed in the AD group, respectively (Figure 5A,B). We observed that these DEGs were enriched in different GO functional categories (Figure 5C).

The DEGs in the AD group were significantly enriched in protein metabolic processes, such as amyloid fibril formation, regulation of amyloid fibril formation, and regulation of amyloid-beta (Aβ) formation. Another significantly enriched category for DEGs was phosphorus metabolic processes, such as regulation of tau-protein kinase activity (*p* < 0.05; Figure 5C), in which the involved DEGs encode Apolipoprotein E (APOE), Clusterin (CLU), and αB-crystallin (CryaB) (Table S4). APOE is thought to be a major genetic risk factor for AD [35]. CLU has been identified as a mediator of Aβ toxicity, and CLU knockdown and knockout have a neuroprotective effect on iPSC-derived neurons in rodents and humans [36]. Moreover, CryaB combines with Aβ, significantly inhibiting their accumulation and neurotoxicity, and the development of AD-like pathology is accompanied by the upregulation of CryaB [37]. 

In addition, clear enrichments were observed in inflammation-related biological processes, including microglial cell activation, positive regulation of inflammatory response, tumor necrosis factor production and regulation, interleukin-6 (IL-6) and interleukin-12 (IL-12) production and regulation, neuron death and regulation, astrocyte activation, NIK/NF-kappaB signaling and regulation, and positive regulation of cytokine production (*p* < 0.05; Figure 5C). In an aged brain, microglial cell activation is more likely to result in a pro-inflammatory phenotype, post-activation phagocytosis is reduced, and amyloid plaques are not removed [38]. Additionally, the high levels of pro-inflammatory cytokines produced (including tumor necrosis factor, IL-6, IL-12, etc.) cause a neuroinflammatory response that kills neurons, resulting in AD [39]. In the AD model, Aβ-induced activation of astrocytes also leads to the release of inflammatory cytokines such as tumor necrosis factor and IL-6 [40]. NF-kappaB activation in astrocytes can boost the expression of several cytokines, and these upregulated cytokines, in turn, activate NF-kappaB, creating a positive feedback system that worsens the inflammatory response and speeds up the degenerative process of AD [41]. There are some DEGs belonging to oligodendrocyte differentiation, myelination, and positive regulation of myelination biological processes (*p* < 0.05; Figure 5C, Table S4). Oligodendrocytes play a crucial role in the formation of myelin in the central nervous system. This can build an insulating myelin layer surrounding axons to aid in the effective jump transmission of bioelectrical signals and preserve and safeguard neurons’ normal function. An abnormal oligodendrocyte can cause demyelination of neurons, neuronal injury, and cause AD [42]. These results indicate that our method can be readily applied to other mouse models of disease, offering a versatile tool to study molecular mechanisms of disease initiation and progression after pathological observation.

## 3. Discussion

In this study, we developed a protocol of unbiased RNA recovery and high-throughput sequencing in PFA-fixed samples and demonstrated that our method achieves comparable performance to fresh samples in terms of gene detection and expression level quantification. Our method will increase the flexibility for researchers in using high-throughput RNA sequencing because PFA fixation has been shown to preserve biomolecules and anatomical structures better in many applications.

Here, we chose phenol–chloroform extraction to purify RNA due to the straightforward protocol, which includes only extraction and precipitation. The reagent used is inexpensive and simple to obtain. The minimal equipment requirements call only for the aid of a centrifuge [43]. The utilization of protease K digestion, followed by a short incubation at an elevated temperature using PBS buffer, has been shown to significantly augment the yield and quality of RNA, as well as enhance the RNA performance in downstream enzymatic assays in fixed tissues. Tissue solubilization and RNA release can be aided by protease K since fixed tissue has a higher hardness. Protease K [44], collagenase [21], pepsin [13], etc., have been used for the digestion of fixed tissues. Temperature, PH, and other variables that can cause protein denaturation are particularly sensitive to collagenase. Pepsin is specific to certain amino acid sequences. The strong activity of protease K in a variety of buffers and a wide range of PH, as well as its broad spectrum, allows it to inactivate nucleases and prevent RNA degradation. In addition to PBS utilized within our method, previous studies have shown that high temperatures and brief incubations in buffers like TE [9], TAE [45], citric acid [46], tris, and phosphate [10] can also reverse the chemical modification generated by cross-linked fixatives, improve RNA yield and quality, and enhance RNA performance in enzyme activities like reverse transcription and PCR. The highly permeable nature of monomeric PFA allows for uniform fixation of large-scale tissues, such as the brain, which in our study had dimensions of 21 × 11 × 11 mm and a larger volume compared to heart, kidney, and lung tissue. The method can also be applied to heart, kidney, and lung tissue with appropriate optimizations in digestion and cross-linking reversal times.

Oligo-dT primer is commonly employed in the production of RNA sequencing libraries. The process of cross-link reversal leads to RNA fragmentation. Consequently, the enrichment of fragmented RNA using oligo-dT primer results in the exclusion of all RNA fragments lacking the polyadenylation site. It has been reported that a mere 27% of reads are uniquely aligned by oligo-dT capture, and only the fragment near the 3′ ends containing the beginning of the polyadenylation tail from each transcript is sequenced [24]. Our results show uniform coverage without 3′ bias in fixed samples, while a discernable 5′ bias is observed for long transcripts (Figure 2C). A 5′ bias does not necessarily imply a problem; it may be viewed as a drop in 3′ coverage due to alternative polyadenylation sites and reverse transcription start sites [47]. Furthermore, due to the presence of miRNA and snoRNA, it is normal to have a certain percentage of reads mapping to intron regions (Figure 2E and Figure S2). Despite the fact that read mapping to intergenic regions was statistically lower in fixed samples (Figure 2E), an intergenic region is a stretch of DNA sequences located between genes that did not form part of the gene structure, which had little effect on our gene expression analyses. It is worth noting that the standard Illumina sequencing requires the use of short cDNA fragment libraries, and our method without fragmentation differs little from the intact RNA using fragmentation during library construction (Figure 2F), which can simply omit the step of fragmentation prior to sequencing.

Various types of genes were detected, and the majority of them were PCGs (Figure 3B), which could perfectly satisfy the needs of gene expression and difference analysis. TEC has polyadenylation features that could indicate the presence of protein-coding genes, but require experimental validation. Pseudogenes have homology to known protein-coding genes but contain a frameshift or stop codon that disrupts the ORF. Fixed samples displayed a larger percentage of TEC and pseudogenes (Figure 3B), which typically have unknown or lost functions and did not significantly hamper downstream molecular analyses. Both PCA and hierarchical cluster analysis (Figure 3D, E) demonstrated a certain extent of separation between fixed and fresh samples, which was consistent with reports in the literature [48,49,50], illustrating that RNA was still affected by PFA fixation even after cross-link reversal, which may be related to the fact that cross-link reversal was incomplete and could not recover the entire RNA. The overall gene expression pattern of fixed samples was close to that of unfixed fresh samples (Figure S3), and the correlation coefficient between PFA-fixed samples and matched fresh samples was higher than that reported in the literature for FFPE samples [23], which further confirmed the impact of paraffin embedding on precise quantification of gene expression level [25]. However, agarose embedding in this work had no discernible effect on the quantity and quality of RNA (Figure S1). It appears to have the same advantages as OCT embedding after PFA fixation for transcriptome research and does not need additional dehydration processing. This could be because agarose solutions do not permeate tissues, and brief immersion at moderately high temperatures is insufficient to trigger RNA loss owing to heat-induced cross-link reversal [51,52].

Furthermore, we applied our methodology to PFA-fixed fluorescence-labeled transgenic mouse brains to investigate specific regions of interest. Discrepancies were observed between the data obtained from fresh tissue [53,54] and our findings within the same brain region. This incongruity may stem from the fact that the data in the cited studies derive from a population of cholinergic neurons within the region, rather than the entirety of the region. Despite the fact that specific regions of fresh samples can be roughly obtained based on Allen Brain Atlas combined with prior knowledge, it is more precise and reliable to obtain a region in accordance with the actual circumstances observed by imaging in each fixed mouse brain section. Fixed samples combined with HE staining can also accurately obtain a region, but additional staining processing cannot avoid adverse effects on RNA [55]. Importantly, it enables efficient sample multiplexing for molecular studies after anatomical and pathological investigations. 

This study has limitations. The tissue size of mouse brain regions is the focus of our research at present. A brain region has numerous cells and an extracellular matrix that collectively contribute to its specialized functions. However, the mechanisms behind intercellular communication and the precise cellular or component determinants accountable for these functions remain unidentified. LCM can be utilized to precisely separate individual cells in place of manual microdissection, which can address the aforementioned issues. Additionally, PFA fixation followed by resin embedding can preserve biological tissues for a longer period of time and provide a more detailed morphological structure of neurons, including the dendritic spines and axonal boutons [56]. In comparison to paraffin, certain acrylic resins exhibit a lower polymerization temperature and have superior molecular analysis capability [57,58]. It is anticipated that our method will be employed for the analysis of resin embedding samples, with the potential to explore gene expression patterns inside specific subcellular regions.

## 4. Materials and Methods

### 4.1. Animals

ChAT-ires-Cre:Ai14 mice (3–5 months, 16–17 months) and 5×FAD:ChAT-ires-Cre:Ai14 transgenic mice (16–17 months) were used in this study. ChAT-ires-Cre mice (stock No: 018957) and Cre-reporter-expressing tdTomato Ai14 mice (stock No: 007914) were obtained from the Jackson Laboratory. ChAT-ires-Cre:Ai14 mice were generated by crossing male ChAT-ires-Cre mice with female tdTomato Cre reporter Ai14 mice. The 5×FAD mouse line expresses the human APP and PSEN1 transgenes, with a total of five FAD mutations: APP KM670/671NL, APP I716V, APP V717I, PSEN1 M146L, and PSEN1 L286V. 5×FAD:ChAT-ires-Cre:Ai14 mice were generated by crossing male 5×FAD mice with female ChAT-ires-Cre:Ai14 mice. Mice were kept in a 12 h light/dark cycle with food and water available ad libitum. All the procedures of animal experiments were approved by the Animal Ethics Committee of the Huazhong University of Science and Technology. All experiments were conducted in accordance with relevant governmental and institutional guidelines for the use of experimental animals. 

### 4.2. Perfusion Fixation

Mice were anesthetized by intraperitoneal injection with 0.1 ml of 1% sodium pentobarbital solution per 10 g weight. After a few minutes or when the mouse no longer responded to painful stimuli, surgical scissors were used to expose the chest cavity. The anesthetized mice were intracardially perfused with 0.01 M phosphate-buffered saline (PBS, Sigma-Aldrich, St. Louis, MO, USA), followed by 4% cold paraformaldehyde (PFA, Sigma-Aldrich, St. Louis, MO, USA) in 0.01 M PBS. The brains were then excised and post-fixed in 4% PFA at 4 °C for at least 24 h.

### 4.3. Microdissection

After fixation, the intact brain was rinsed overnight at 4 °C in 0.01 M PBS. The PFA-fixed brain samples were glued to the sample base of the vibrating slicer (VT 1200S, Leica, Wetzlar, Germany) and filled with 0.01 M PBS. By adjusting the amplitude to 1.0 mm and the slicing speed to 1.5 mm/s, the brain was sectioned into 70 µm coronal slices. The acquisition of coronal slices was assisted by agarose embedding, which has been previously described [59]. All sections were collected for further imaging with a confocal microscope (LSM 710, Zeiss, Jena, Germany). All sections containing MS/VDB, XII, and mPFC were selected for microdissection according to the tdTomato signal and the Allen Reference Brain Atlas. For control experiments, fresh, intact brain samples were filled with 0.01 M PBS in an ice-water mixed state during sectioning. By lowering the slicing speed to 0.24 mm/s, the same areas were dissected. All steps were performed under RNase-free conditions.

### 4.4. RNA Isolation

Briefly, 40 U/mL Proteinase K (Qiagen, Hilden, Germany) in 0.01 M PBS buffer was added to the microdissected tissue sample and incubated at 55 °C for 1 h. The temperature was increased to 70 °C for 15 min to reverse cross-links. Amounts of 500 μL of TRIzol (Invitrogen, Waltham, MA, USA) and 100 μL chloroform (Sinopharm Chemical Reagent Co., Ltd., Shanghai, China) were added, gently vortexed, and the sample was centrifuged at 12,000× *g* for 15 min at 4 °C. The colorless upper aqueous phase containing the RNA was transferred to a new tube without contamination. A 1/10 volume of 3 M sodium acetate (Invitrogen, Waltham, MA, USA), 1 μL of 20 mg/mL glycogen (Thermo Fisher Scientific, Waltham, MA, USA) per 20 μL of the solution, and 2.5 volumes of ethanol (Sigma-Aldrich, St. Louis, MO, USA) were added, mixture vortexed, and incubated at −70 °C for 30 min. The mixture was centrifuged at 12,000× *g* for 10 min at 4 °C, the supernatant was discarded, and the pellet was rinsed three times with cold 70% ethanol. After the pellet was air-dried, the total RNA pellet was dissolved in nuclease-free water. All tubes and tips were RNase-free, and all reagents were of molecular biology grade if available when working with RNA. For fresh brain samples, RNA isolation using TRIzol (Invitrogen, Waltham, MA, USA) was based on the conventional phenol–chloroform extraction principle according to the manufacturer’s instructions. RNA yield was measured using the Nanodrop One UV Spectrophotometer (Thermo Fisher Scientific, Waltham, MA, USA) and Qubit 4 Fluorometer (Invitrogen, Waltham, MA, USA). The RNA recovery rate was calculated on the basis of the real RNA yield of the fresh sample. RNA quality, classified by RIN and DV200, was determined with the 2100 Bioanalyzer RNA 6000 Pico Chip (Agilent Technologies, Santa Clara, CA, USA). 

### 4.5. Library Construction and Sequencing

Genomic DNA (gDNA) contamination was removed using a DNase I (Qiagen, Hilden, Germany). The RiboZero beads from TruSeq^®^ Stranded Total RNA Library Prep Gold (Illumina, San Diego, CA, USA) were used to deplete rRNA and mtrRNA from total RNA. Fragmentation was carried out by incubating at 94 °C for 15 min in First-Strand Synthesis Reaction Buffer from the NEBNext^®^ Ultra™ Directional RNA Library Prep Kit for Illumina (New England Biolabs, Ipswich, MA, USA), only for intact mRNA from fresh samples. First-strand cDNA was synthesized using random primer and ProtoScript II Reverse Transcriptase. Second-strand cDNA synthesis was subsequently performed, and the double-stranded cDNAs were purified with 1.8× Agencourt AMPure XP Beads (Beckman Coulter, Indianapolis, IN, USA). After the ends were repaired, adenylation of 3′ terminals of cDNA was performed and adaptors were added, and adaptor-ligated cDNA with an insert size of about ~300 bp were size-selected with the AMPure XP system. Then, after 15 cycles of PCR amplification, the PCR product was purified with 0.9× Agencourt AMPure XP beads again, and the library was finally obtained. The library was quantified by Qubit 4 Fluorometer (Invitrogen, Waltham, MA, USA), and the size and quality of the libraries were assessed in a 2100 Bioanalyzer DNA High Sensitivity Chip (Agilent Technologies, Santa Clara, CA, USA). After the library was qualified, paired-end 150 base pair sequencing was performed on an Illumina NovaSeq 6000 (Illumina, San Diego, CA, USA).

### 4.6. Read Alignment

Reads were quality assessed and filtered by running fastp (v0.19.7) on the fastq files. Reference genome (http://ftp.ensembl.org/pub/release-104/fasta/mus_musculus/, accessed on 1 August 2023) and gene model annotation files (http://ftp.ensembl.org/pub/release-104/gtf/mus_musculus/, accessed on 1 August 2023) were downloaded from genome database directly. The index of the reference genome was built using HISAT2 [60] (v2.0.5), and paired-end clean reads were aligned to the mouse reference genomes using HISAT2 with the corresponding Ensembl GRCm38 genome. SAMtools View (v1.16.1) was used to convert SAM files to BAM files, and BAM files were sorted and indexed using SAMtools Sort (v1.16.1) and SAMtools Index [61,62] (v1.16.1). The Gene Body Coverage module in RSeQC [63] (v4.0.0) was used to calculate read coverage over 5′ to 3′ gene bodies. The Read Distribution module in RSeQC was used to calculate how mapped reads were distributed over genome features, like exon, intron, and intergenic regions. When genome features overlapped, they were prioritized as follows: exons > introns > intergenic regions. The read alignment was visually explored at different scales using IGV [64] (v2.17.1). The Inner Distance module in RSeQC was used to calculate the inner distance (or insert size) between two paired reads.

### 4.7. Gene Expression Quantification

FeatureCounts [65] (v1.5.0) was used to count the read numbers mapped to each gene. The matrix of read counts and the design file were imported to R. For each sample, read counts were divided by the total number of mapped reads and multiplied by one million to obtain counts per million (CPM) to account for varying library sizes. Each gene expression level was calculated based on FPKM, which was calculated by dividing the CPM values by the gene lengths [66]. Genes with FPKM values of zero across all samples were removed, and the gene count was generated according to whether its FPKM was greater than 0 in the sample. The resampling method in RSeQC was used to check whether the current sequencing depth was saturated or not (or if the FPKM values were stable or not) by using subsets of the reads data.

### 4.8. Analysis of Gene Expression Differences

The DESeq2 R package (v1.20.0) [67] was used to screen for area-dependent differentially expressed genes, which directly handles the raw read count data. Brain-area-specific genes were identified using different FC thresholds of 2, 4, 8, 16, and 32 (Supplementary Table S2). A gene was considered to be an area-specific gene if its expression level was more than 8-fold higher in a given brain area over any other two areas. Differential gene expression in the MS/VDB area of 5 × FAD mice was also analyzed with DESeq2. The threshold value for identifying DEGs was defined as an FC cutoff of 2. The GO database (http://geneontology.org/, accessed on 15 October 2023) provides functional categorization and annotations for large-scale transcriptomic data. Functional annotation was conducted using the clusterProfiler R package (v3.8.1) [68]. The significantly enriched results were selected with the following cutoff: adjusted *p*-value less than 0.05. 

## 5. Conclusions

We developed and validated the method of reliable sequencing and accurate gene expression analysis in PFA-fixed tissue. Our method is of great value in advancing our understanding of biological systems at multiple levels. We anticipate that whole transcriptome-wide gene expression profiling in PFA-fixed biological samples will be widely adopted in diverse areas and will pave the way for new discoveries in the field of biology. 

## Data Availability

All datasets generated or analyzed during this study are available from the corresponding author on request.

## Supplementary Materials

The following supporting information can be downloaded at: https://www.mdpi.com/article/10.3390/ijms25126504/s1.
