# Supplementary material for: Streamlined Full-Length Total RNA Sequencing of Paraformaldehyde-Fixed Brain Tissues
Source: Int J Mol Sci. 2024 Jun 13;25(12):6504. doi: 10.3390/ijms25126504 (PMC11204141; doi:10.3390/ijms25126504)
Supplement: Supplementary file 1 [file ijms-25-06504-s001.zip › ijms-3005799-supplementary.pdf]

Supplementary Materials

Supplementary Figures

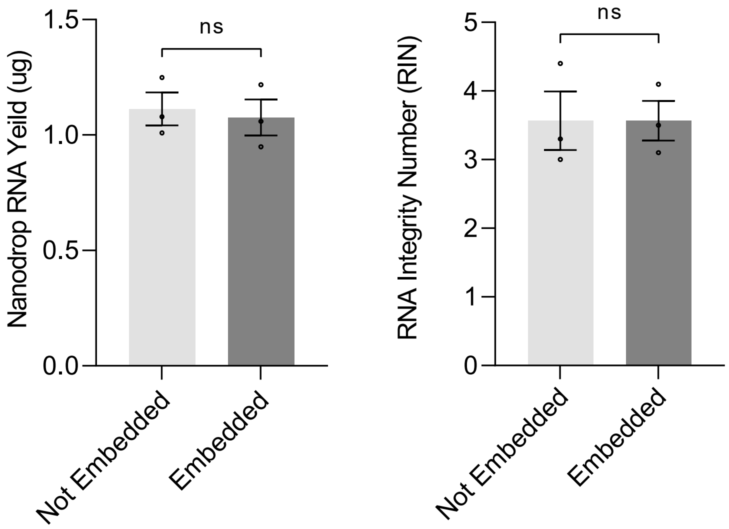

Figure S1. Effects of agarose embedding on RNA quantity and quality. Short-term embedding of PFA-fixed sample did not impair RNA quantity (1.08±0.08ug) and RIN values (3.6±0.3) compared with not embedding (1.11±0.07ug and 3.6±0.4, respectively).

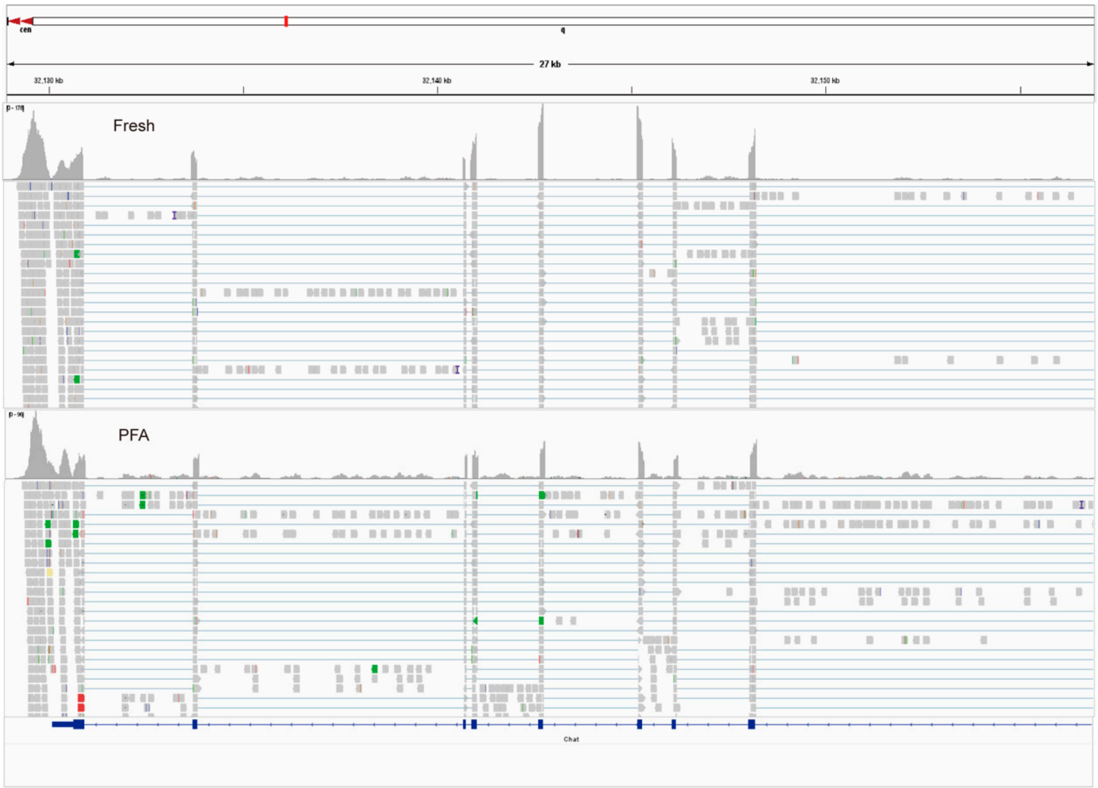

Figure S2. Reads of Fresh and PFA groups visualized on the IGV. Location on the genome is indicated by the horizontal axis, and the number of reads is shown by the vertical axis. The top portion shows an ideogram of chromosome 14 with a vertical red bar at 2,128,918-32,156,894bp. The bottom portion shows the exon region (blue rectangle) and intron region (blue line). In this example, a large fraction of reads are mapped to the exon regions.

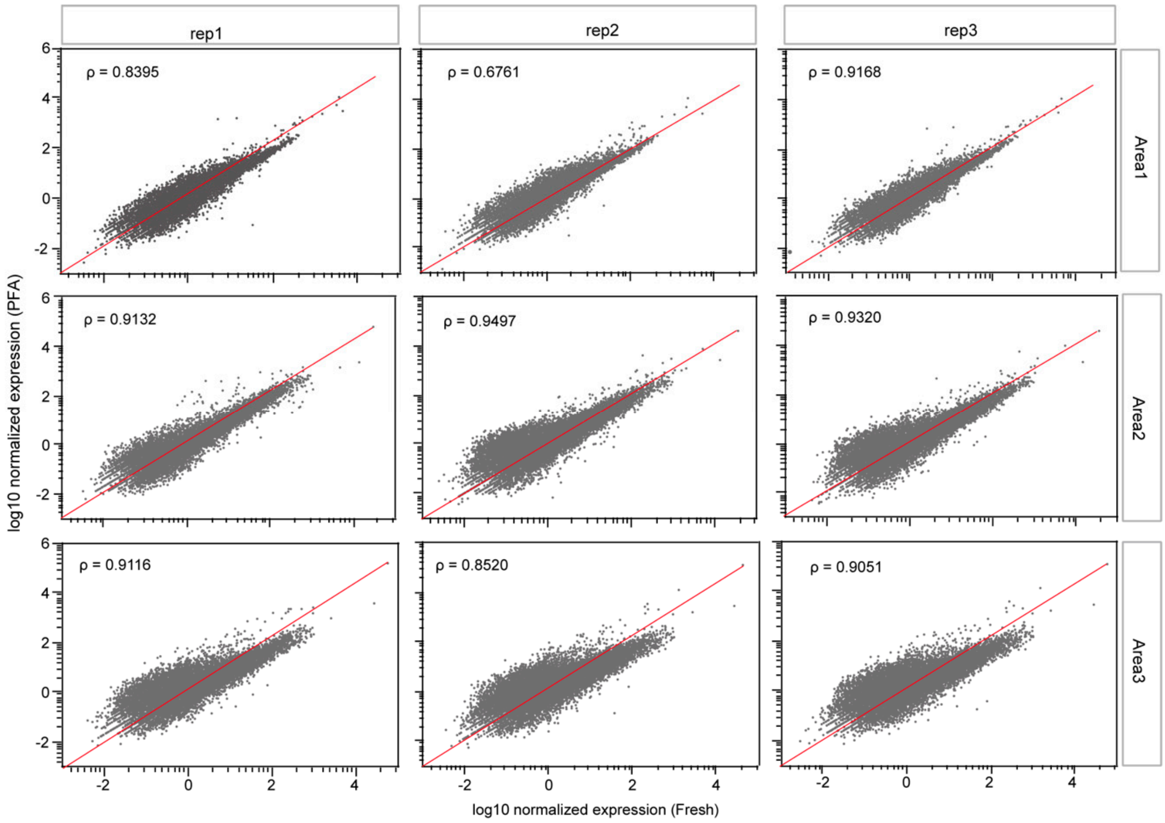

Figure S3. Comparison of the log-normalized expression level of each gene between the PFA and Fresh groups. Each dot represents the relative expression level of a gene, and the red line indicates the line y = x. The plots also show the Pearson's correlation coefficient ρ of the log-normalized expression level between fixed and fresh tissues for three brain areas.

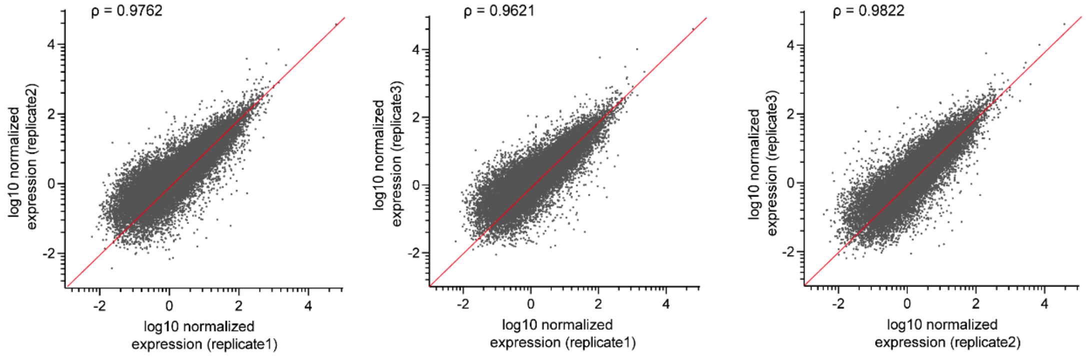

Figure S4. Scatter plot showing three technical replicates in PFA-fixed samples. Each dot represents the relative normalized expression level, and the red line indicates y = x. The plot also shows Pearson's correlation coefficient ρ of the log-normalized expression levels between the two replicates.

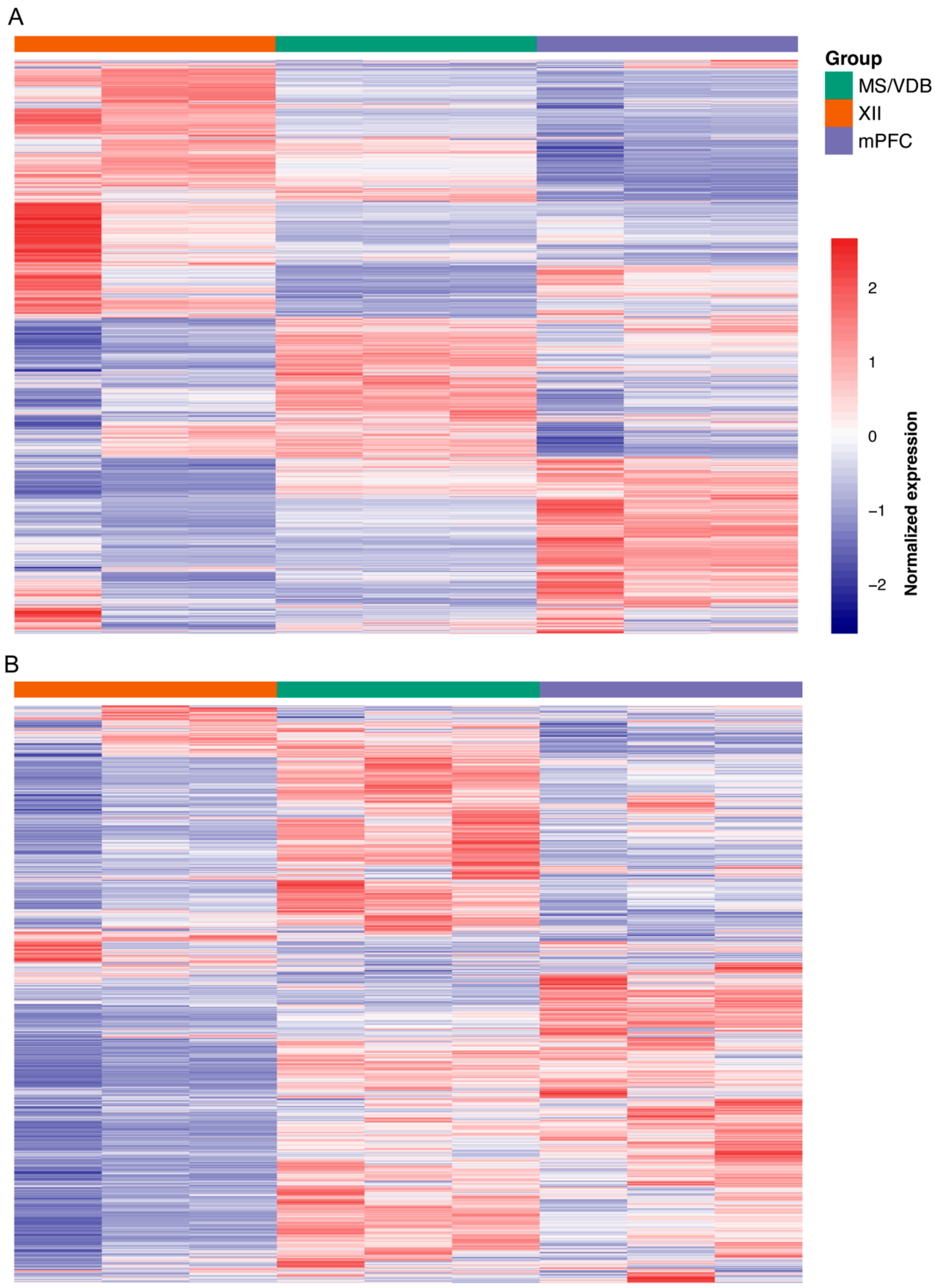

Figure S5. Hierarchical clustering analysis of gene expression profiles of 9 samples with protein-coding genes (A) and ncRNA genes (B). Each column indicated a sample, whereas each row indicated a gene. Each brain area symbol was shown on the color bar for each cluster.

Supplementary Tables

Table S1. Comparison of present method with selected similar RNA-seq workflows with different tissue preservation methods. Analogous steps are aligned in the same row. RT: reverse transcription. TS-RT: template-switching reverse transcription.

Tissue preparation	fixed	fixed	fixed
	dehydration	dehydration	
	OCT embedding	paraffin embedding	agarose embedding
RNA isolation		paraffin removal	
	pepsin lysis	pK lysis	pK lysis
	removing cross-links with TE	removing cross-links with TE	removing cross-links with PBS
	RNA purification	RNA purification	RNA purification
Library construction	Poly(A) selection		Ribosomal RNA depletion
	TS-RT, oligo(dT)-primed	TS-RT, random-primed	First-strand RT, random-primed
	Second-strand synthesis	Second-strand synthesis	Second-strand synthesis
		Adaptor Ligation	
	Library PCR	Library PCR	
	Cleanup & size-selection	Cleanup & size-selection	
		Ribosomal cDNA depletion	
	cDNA fragmentation		
	Adaptor Ligation		Adaptor Ligation
	Cleanup & size-selection		Cleanup & size-selection
	Library PCR	Library PCR	Library PCR
	Cleanup & size-selection	Cleanup & size-selection	Cleanup & size-selection

Table S2. Number of brain region-specific genes using different FC thresholds.

**Brain area**	**Fold Change (FC)**				
	**2**	**4**	**8**	**16**	**32**
**MS/VDB**	802	167	51	28	16

**XII**	2093	393	111	53	34
**mPFC**	1532	462	153	61	27

Table S3. The detailed information of top 20 GO enrichment terms (adjusted p < 0.05) for brain region-dependent differentially expressed genes in MS/VDB, XII, mPFC, respectively.

	**Category**	**GOID**	**Description**	**adjusted p**	**geneName**	**Count**
Top 20 GO terms enrichment of the MS/VDB-dependent differentially expressed genes	BP	GO:0007623	circadian rhythm	2.70E-07	Six3/Nkx2-1/Drd3/Prok2/Ngfr/Mc3r/Drd1/Adora2a	8
	BP	GO:0008015	blood circulation	3.26E-06	Nkx2-1/Drd3/Scn5a/Adra2b/Mc3r/Drd1/Adora2a/Kcnj5/Postn	9
	BP	GO:0007626	locomotory behavior	0.00010969	Nkx2-1/Drd3/Gbx1/Mc3r/Drd1/Adora2a	6
	BP	GO:0051966	regulation of synaptic transmission, glutamatergic	0.00040374	Drd3/Ngfr/Drd1/Adora2a	4
	BP	GO:0050795	regulation of behavior	0.00078859	Drd3/Mc3r/Drd1/Adora2a	4
	BP	GO:0045187	regulation of circadian sleep/wake cycle, sleep	0.00078859	Drd3/Drd1/Adora2a	3
	BP	GO:0008217	regulation of blood pressure	0.00078859	Nkx2-1/Drd3/Adra2b/Mc3r/Postn	5
	BP	GO:0071875	adrenergic receptor signaling pathway	0.00080668	Drd3/Adra2b/Drd1	3
	BP	GO:0035249	synaptic transmission, glutamatergic	0.00080668	Drd3/Ngfr/Drd1/Adora2a	4
	BP	GO:0001963	synaptic transmission, dopaminergic	0.00080668	Drd3/Drd1/Adora2a	3
	BP	GO:0001975	response to amphetamine	0.00097852	Drd3/Drd1/Adora2a	3
	BP	GO:1903522	regulation of blood circulation	0.00108871	Scn5a/Adra2b/Mc3r/Drd1/Kcnj5	5
	BP	GO:0007631	feeding behavior	0.0011613	Nkx2-1/Mc3r/Drd1/Adora2a	4
	BP	GO:0030431	sleep	0.00138865	Drd3/Drd1/Adora2a	3
	BP	GO:0007189	adenylate cyclase-activating G-protein coupled receptor signaling pathway	0.0014335	Drd3/Mc3r/Drd1/Adora2a	4
	BP	GO:0006936	muscle contraction	0.00208199	Prok2/Scn5a/Adra2b/Drd1/Kcnj5	5
	BP	GO:0055078	sodium ion homeostasis	0.00254276	Drd3/Mc3r/Adora2a	3
	BP	GO:0097366	response to bronchodilator	0.0025718	Drd3/Drd1/Adora2a	3
	BP	GO:0021879	forebrain neuron differentiation	0.0025718	Lhx8/Nkx2-1/Drd1	3
	BP	GO:0032224	positive regulation of synaptic transmission,	0.0025718	Ngfr/Adora2a	2

			cholinergic			
Top 20 GO terms enrichment of the XII-dependent differentially expressed genes	BP	GO:0003002	regionalization	6.16E-09	Hoxb5/Hoxa5/Hoxc4/Hoxb6/Hoxc5/Hoxb3/Tbx20/Hoxb4/Hoxa3/Pax8/Irx3/Lhx1/Irx2/Gbx2/Irx1/Foxj1	16
	BP	GO:0048706	embryonic skeletal system development	1.57E-08	Hoxb5/Hoxa5/Hoxc4/Hoxb6/Hoxc5/Hoxb3/Hoxb4/Hoxa3/Shox2/Irx5/Lhx1	11
	BP	GO:0072006	nephron development	4.48E-08	Pax8/Pax2/Irx3/Lhx1/Irx2/Cd24a/Cited1/Ret/Irx1/Foxj1/Pgf	11
	BP	GO:0048562	embryonic organ morphogenesis	3.72E-07	Hoxb5/Hoxa5/Hoxc4/Hoxb6/Hoxb3/Tbx20/Hoxb4/Hoxa3/Pax8/Shox2/Irx5/Lhx1/Gbx2	13
	BP	GO:0048705	skeletal system morphogenesis	1.63E-06	Hoxb5/Hoxa5/Hoxc4/Hoxb6/Hoxb3/Hoxb4/Hoxa3/Shox2/Irx5/Lhx1/Acan	11
	BP	GO:0009952	anterior/posterior pattern specification	5.37E-06	Hoxb5/Hoxa5/Hoxc4/Hoxb6/Hoxc5/Hoxb3/Hoxb4/Hoxa3/Lhx1/Gbx2	10
	BP	GO:0001763	morphogenesis of a branching structure	6.97E-06	Hoxa5/Tbx20/Pax8/Shox2/Pax2/Vdr/Lhx1/Cited1/Gbx2/Pgf	10
	BP	GO:0001655	urogenital system development	1.75E-05	Pax8/Pax2/Irx3/Spp1/Lhx1/Irx2/Cd24a/Cited1/Ret/Irx1/Foxj1/Pgf	12
	BP	GO:0030858	positive regulation of epithelial cell differentiation	2.96E-05	Pax8/Vdr/Lhx1/Cd24a/Foxj1/Trim16	6
	BP	GO:0001657	ureteric bud development	2.96E-05	Pax8/Pax2/Lhx1/Cited1/Ret/Foxj1/Pgf	7
	BP	GO:0021953	central nervous system neuron differentiation	3.51E-05	Otp/Tbx20/Phox2b/Nkx6-1/Lhx4/Lhx1/Lhx5/Nhlh2/Gbx2	9
	BP	GO:2000177	regulation of neural precursor cell proliferation	5.46E-05	Slc6a4/Otp/Lhx1/Cd24a/Lhx5/Gjc2/Trf	7
	BP	GO:0030878	thyroid gland development	0.000390532	Hoxa5/Hoxb3/Hoxa3/Pax8	4
	BP	GO:0072182	regulation of nephron tubule epithelial cell differentiation	0.000661464	Pax8/Lhx1/Cd24a	3
	BP	GO:0061564	axon development	0.00072443	Phox2b/Shox2/Nkx6-1/Lhx4/Chodl/Lhx1/Nkx2-9/Ret/Nefh/Gbx2/Apod	11
	BP	GO:0048265	response to pain	0.000729896	Calca/Dbh/Ret/Gch1	4
	BP	GO:0030902	hindbrain development	0.000771467	Slc6a4/Hoxb3/Phox2b/Lhx1/Lhx5/Nhlh2/Gbx2	7
	BP	GO:0061351	neural precursor cell proliferation	0.000771467	Slc6a4/Otp/Cd24a/Lhx5/Gbx2/Gjc2/Trf	7
	BP	GO:0021936	regulation of cerebellar granule cell precursor proliferation	0.001231926	Slc6a4/Lhx1/Lhx5	3
	BP	GO:0042428	serotonin metabolic process	0.00148175	Tph2/Gch1/Ddc	3

Top 20 GO terms enrichment of the mPFC-dependent differentially expressed genes	BP	GO:0050890	cognition	6.89E-08	Tbr1/Slc17a7/Neurod2/Chrm1/Nrgn/Synpo/Ptgs2/Cck/Rin1/Rgs14/Prkcg/Itga8/Adcy1/Nptx2/Shank1/Npas4/Slc8a2	17
	BP	GO:0048167	regulation of synaptic plasticity	6.89E-08	Itpka/Neurod2/Nrgn/Synpo/Camk2a/Ptgs2/Rin1/Rgs14/Ptk2b/Prkcg/Adcy1/Shank1/Npas4/Slc8a2	14
	BP	GO:0007611	learning or memory	6.89E-08	Tbr1/Slc17a7/Neurod2/Nrgn/Synpo/Ptgs2/Cck/Rin1/Rgs14/Prkcg/Itga8/Adcy1/Nptx2/Shank1/Npas4/Slc8a2	16
	BP	GO:0008306	associative learning	1.20E-05	Tbr1/Neurod2/Nrgn/Synpo/Cck/Rin1/Rgs14/Nptx2/Shank1	9
	BP	GO:0050806	positive regulation of synaptic transmission	3.10E-05	Stx1a/Nrgn/Ptgs2/Rgs14/Ptk2b/Prkcg/Adcy1/Shank1/Cacnb3/Cckbr/Slc8a2	11
	BP	GO:0060078	regulation of postsynaptic membrane potential	0.00015038	Slc17a7/Stx1a/Chrm1/Ptk2b/Gabrd/Shank1/Npas4/Cacnb3/Slc8a2	9
	BP	GO:0030900	forebrain development	0.00070182	Satb2/Emx1/Fezf2/Neurod6/Tbr1/Rtn4rl2/Mas1/Rtn4r/Lhx2/Nrgn/Sstr4/Wnt4/Rtn4rl1	13
	BP	GO:0035249	synaptic transmission, glutamatergic	0.0008798	Slc17a7/Ptgs2/Grm2/Ptk2b/Shank1/Dgkz/Cckbr	7
	BP	GO:0060291	long-term synaptic potentiation	0.00090927	Nrgn/Rgs14/Ptk2b/Prkcg/Adcy1/Shank1/Slc8a2	7
	BP	GO:0007616	long-term memory	0.00104762	Slc17a7/Rgs14/Adcy1/Shank1/Npas4	5
	BP	GO:0060079	excitatory postsynaptic potential	0.00104959	Slc17a7/Stx1a/Ptk2b/Shank1/Npas4/Cacnb3/Slc8a2	7
	BP	GO:0051966	regulation of synaptic transmission, glutamatergic	0.00104959	Ptgs2/Grm2/Ptk2b/Shank1/Dgkz/Cckbr	6
	BP	GO:0050808	synapse organization	0.00134053	Igfn1/Itpka/Neurod2/Pdzrn3/Synpo/Shank1/Npas4/Dlgap3/Plxnd1/Cacnb3/Dgkz/Slc8a2/Bsn	13
	BP	GO:0099565	chemical synaptic transmission, postsynaptic	0.00143033	Slc17a7/Stx1a/Ptk2b/Shank1/Npas4/Cacnb3/Slc8a2	7
	BP	GO:0021537	telencephalon development	0.00188931	Emx1/Fezf2/Neurod6/Tbr1/Rtn4rl2/Mas1/Rtn4r/Lhx2/Nrgn/Rtn4rl1	10
	BP	GO:0043087	regulation of GTPase activity	0.00210367	Rtn4r/Wnt4/Rin1/Rgs14/Ptk2b/Iqgap2/Agap2/Ccl19/Plxnd1/Stard8/Rasal1/Arhgap25	12
	BP	GO:0021877	forebrain neuron fate commitment	0.00296254	Satb2/Fezf2/Tbr1	3
	BP	GO:0098693	regulation of synaptic vesicle cycle	0.00319634	Slc17a7/Stx1a/Camk2a/Cplx3/Prkcg/Adcy1/Bsn	7
	BP	GO:0050848	regulation of calcium-mediated signaling	0.00319634	Neurod2/Jsrp1/Ptk2b/Itpr1/Fhl2/Slc8a2	6
	BP	GO:0042391	regulation of membrane potential	0.00319634	Slc17a7/Stx1a/Kcnh3/Chrm1/Cck/Ptk2b/Kcnh4/Gabrd/Shank1/Npas4/Cacnb3/Slc8a2	12

Table S4. The detailed information of GO enrichment terms (adjusted $p < 0.05$) for differentially expressed genes between the AD and Control samples.

Category	GOID	Description	adjusted p	geneName	Count
BP	GO:0031644	regulation of neurological system process	5.38E-05	Cst7/Fabp5/Itgax/Ctss/S100b/Nmu/Cartpt/Trf	8
BP	GO:0001774	microglial cell activation	9.26E-05	Trem2/Cst7/Tyrobp/C1qa/Clu/Aif1	6
BP	GO:0061900	glial cell activation	0.00018679	Trem2/Cst7/Tyrobp/C1qa/Clu/Aif1	6
BP	GO:0031643	positive regulation of myelination	0.00078951	Cst7/Itgax/S100b/Trf	4
BP	GO:0042063	gliogenesis	0.0011135	Gfap/Trem2/C1qa/Clu/S100b/Vtn/Slc45a3/Cd9/Mdk/Trf	10
BP	GO:0042116	macrophage activation	0.00134477	Trem2/Cst7/Tyrobp/C1qa/Clu/Aif1	6
BP	GO:0042552	myelination	0.00134477	Cst7/Bcas1/Itgax/Clu/S100b/Cd9/Trf	7
BP	GO:0008366	axon ensheathment	0.00134477	Cst7/Bcas1/Itgax/Clu/S100b/Cd9/Trf	7
BP	GO:0048102	autophagic cell death	0.00182382	Trem2/Ctsl/Laptm5	3
BP	GO:1905906	regulation of amyloid fibril formation	0.00283779	Apoe/Clu/Cryab	3
BP	GO:0050729	positive regulation of inflammatory response	0.0035882	Ctss/Ddt/Nfkbia/Mdk/Rps19/Clec7a	6
BP	GO:0001819	positive regulation of cytokine production	0.00436228	Trem2/Tyrobp/Clu/Laptm5/Ddx3x/Aif1/Ddt/Mdk/Chil1/Clec7a	10
BP	GO:1900221	regulation of amyloid-beta clearance	0.00436228	Trem2/Apoe/Clu	3
BP	GO:1901222	regulation of NIK/NF-kappaB signaling	0.00661946	Trem2/Calr/Laptm5/Ddx3x/Chil1	5
BP	GO:0032640	tumor necrosis factor production	0.00661946	Trem2/Gpnmb/Tyrobp/Clu/Ddt/Clec7a	6
BP	GO:0032680	regulation of tumor necrosis factor production	0.00661946	Trem2/Gpnmb/Tyrobp/Clu/Ddt/Clec7a	6
BP	GO:0048709	oligodendrocyte differentiation	0.00778657	Clu/Vtn/Slc45a3/Cd9/Mdk	5
BP	GO:1990000	amyloid fibril formation	0.00778657	Apoe/Clu/Cryab	3
BP	GO:0038061	NIK/NF-kappaB signaling	0.00802545	Trem2/Calr/Laptm5/Ddx3x/Chil1	5
BP	GO:0097242	amyloid-beta clearance	0.00887158	Trem2/Apoe/Clu	3

BP	GO:1901214	regulation of neuron death	0.00944482	Gpnmb/Tyrobp/Apoe/C1qa/Clu/Ctsz/Psmc1/Mdk	8
BP	GO:0070997	neuron death	0.01496219	Gpnmb/Tyrobp/Apoe/C1qa/Clu/Ctsz/Psmc1/Mdk	8
BP	GO:0032635	interleukin-6 production	0.01990139	Trem2/Tyrobp/Laptm5/Aif1/Clec7a	5
BP	GO:0032675	regulation of interleukin-6 production	0.01990139	Trem2/Tyrobp/Laptm5/Aif1/Clec7a	5
BP	GO:1902947	regulation of tau-protein kinase activity	0.02358329	Apoe/Clu	2
BP	GO:0032615	interleukin-12 production	0.03121285	Laptm5/Mdk/Clec7a	3
BP	GO:0032655	regulation of interleukin-12 production	0.03121285	Laptm5/Mdk/Clec7a	3
BP	GO:1902004	positive regulation of amyloid-beta formation	0.04340651	Apoe/Clu	2
BP	GO:1902430	negative regulation of amyloid-beta formation	0.04340651	Apoe/Clu	2
BP	GO:0048143	astrocyte activation	0.04944724	Trem2/C1qa	2
